# Non-Dairy Animal Protein Consumption Is Positively Associated with Overweight and Obesity in Israeli Adolescents

**DOI:** 10.3390/foods11142072

**Published:** 2022-07-12

**Authors:** Chen Dor, Aliza Hannah Stark, Rita Dichtiar, Lital Keinan-Boker, Tali Sinai

**Affiliations:** 1 Israel Center for Disease Control, Israel Ministry of Health, Ramat Gan 5262100, Israel; hen.dor@moh.gov.il (C.D.); rita.dichtiar@moh.gov.il (R.D.); lital.keinan2@moh.gov.il (L.K.-B.); 2 School of Nutritional Sciences, The Robert H. Smith Faculty of Agriculture, Food and Environment, The Hebrew University of Jerusalem, Rehovot 7610001, Israel; aliza.stark@mail.huji.ac.il; 3 School of Public Health, University of Haifa, Haifa 3498838, Israel

**Keywords:** youth, BMI, Israel, dietary intake, protein

## Abstract

Protein consumption apparently plays a role in weight control. This cross-sectional study examined the association of protein consumption in Israeli adolescents with overweight/obesity. 7th–12th grade students participating in a national school-based survey (2015–2016) completed self-administered questionnaires, including a food frequency questionnaire, and height and weight measurements (*n* = 3443, 48% males, 15.2 ± 1.6 years). WHO growth standards served to define weight status. Intakes of total protein and protein source were calculated. Multivariable logistic regression analyses evaluated associations with overweight/obesity (BMI z-score ≥ 1), adjusting for possible covariates. Total protein intake (median (IQR)) was 62.5 (45.5, 85.7) g/d, accounting for 12.0 (10.5, 13.6) percent of daily energy. Of participants, 31.4% were overweight/obese. In multivariable models, overweight/obesity was positively associated with incremental increases of 10 g/d in total protein intake (OR = 1.07, 95% CI: 1.02–1.12, *p* < 0.01), total animal protein intake (OR = 1.05, 95% CI: 1.01–1.10, *p* = 0.026), and non-dairy animal protein intake (OR = 1.06, 95% CI: 1.01–1.11, *p* = 0.029). No associations were found with plant or dairy protein intake. These associations remained when protein intake was reported as a percentage of daily energy and when overweight and obesity were analyzed individually. High daily protein intakes, principally from non-dairy animal sources, were positively associated with overweight/obesity in adolescents. Additional studies are needed to establish causality of these findings.

## 1. Introduction

Worldwide, high rates of overweight and obesity present a major public health problem. This is of particular concern among children and adolescents. The World Health Organization (WHO) present data indicating that the global prevalence of overweight and obesity among children and adolescents aged 5–19 is over 18% with an estimated 340 million children and adolescents affected [1]. In Israel, a higher prevalence has been reported, with about 30% of Israeli adolescents aged 12–18 diagnosed as overweight/obese. Without substantial intervention, the prevalence is expected to rise in the coming years [2].

Childhood and adolescent overweight and obesity increase the odds of becoming an adult with overweight or obesity [3,4], and can result in serious long-term health and social consequences. Excess weight is often the source of psychological problems [5,6], increased socioeconomic burden [7,8], and comorbidities such as type 2 diabetes mellitus, cardiovascular diseases, and non-alcoholic fatty liver disease (NAFLD) [8,9], all of which impact mortality later in life [10]. Preventing excess weight is, therefore, critical for maintaining physiological and psychological health and preventing premature death.

Obesity during adolescence is the result of complex interactions of behavioral and biological factors that, in general, lead to an increase in energy intake relative to energy expenditure [11]. This positive energy balance results in the storage of energy, primarily as adipose tissue [12]. Dietary patterns have been spotlighted as modifiable lifestyle factors that may impact the development of overweight/obesity, especially in children and adolescents. The amount of food consumed, food source, energy density, and the specific metabolic contribution of specific energy-yielding macronutrients (fats, protein, and carbohydrates) are considered to have a major impact on body weight [13].

Protein is an essential component of the daily diet, and is of particular necessity for normal, somatic growth and development of children and adolescents [14]. In Western diets, the role of protein in weight management is highly controversial and both quantity and source of protein are thought to impact metabolism. On the one hand, protein increases thermogenesis and satiety more than other macronutrients. The literature indicates that in adults, high-protein diets are beneficial for the prevention of weight gain and for weight maintenance [15,16]. On the other hand, studies focusing on overall protein intake during childhood and adolescence, especially in infancy and early childhood, have shown that high protein intake may increase the risk of obesity in late childhood or adulthood [17,18,19,20,21].

Only a few studies in the recent literature explored associations between protein consumption and overweight/obesity among adolescents, with no decisive results [22,23,24]. The paucity of data examining the effect of different protein sources (animal or plant) does not allow for clear conclusions concerning the impact of protein source on body weight. Thus, the aim of this study was to examine the association between intake of total dietary protein, in total and by protein source (plant, dairy, and non-dairy animal protein) among Israeli adolescents with overweight/obesity, based on health and nutrition data collected in a recent national survey.

## 2. Material and Methods

### 2.1. Study Procedures and Sampling

In this study, we used data from the 2nd Israeli Youth Health and Nutrition Survey (MABAT TZAIR 2). The survey design, sampling method, and data sources have been described in detail elsewhere [25,26,27]. Briefly, this was a cross-sectional, nationally representative survey, carried out in schools, including 7th to 12th grade students. It was reviewed and approved by the Ethics Committee of the Sheba Medical Center, and adhered to the Ministry of Education requirements. The compliance rate of the schools was high: 217/234 (93%), as well as of the students 5589/7029 (80%). Participating students were given a self-administered questionnaire to complete, which included demographics, health status, and food frequency intake information. The entire questionnaire is available electronically [25]. Anthropometric measurements were conducted by trained study staff. The students were informed that they reserved the right to refuse to participate at any time during the survey [25].

Data of 3443 students were included in the current analyses. Those who did not complete study questionnaires (*n* = 1683) and/or measurements (*n* = 595) were excluded. Underweight participants (*n* = 56) were also excluded to allow for dichotomization of weight status to overweight/obese versus normal.

### 2.2. Participants Characteristics

Data were collected from survey responses (age, sex, dietary intake, and physical activity), or based on the Israeli Ministry of Education information (population group (Jewish/Arab) and socioeconomic status (SES) which was defined by school welfare level). SES was categorized to low or medium–high, as reported previously [26,27]. Physical activity (PA) was recorded as previously described [26,27]. Participants who performed any PA (walking, cycling, running, swimming, ball games, etc.) for at least an hour a day, on average, were classified as “physically active as recommended” [28].

### 2.3. Dietary Evaluation

The habitual food consumption of the participants was evaluated using a semi-quantitative food frequency questionnaire (FFQ), that included 103 food items commonly eaten in Israel, standard portion sizes, and a frequency response section [25]. The questionnaire has been described in detail elsewhere [25,26,27]. It is based on a validated FFQ used for determining dietary intake of Israeli multiethnic populations [29]. In addition, it was assessed by a national steering committee and minor modifications were made to accommodate for use in adolescents. The adopted questionnaire was used in both the first and second Israeli MABAT Youth surveys. To ensure accuracy, a subsample of 467 (12%) of participants were interviewed using 24 h dietary recall to calibrate the energy and macronutrient values generated from the FFQ [25].

Total energy (Kcal) and macronutrient intakes (grams) were calculated based on the Israeli food and nutrient database using the Tzameret software [30]. The distribution of macronutrients as a percentage of total daily energy intake was also calculated and compared to dietary reference intake (DRI) values [31]. Protein intake was categorized into 3 subtypes: plant protein (grains, nuts, legumes, vegetables, and fruits), dairy protein (items that were produced from mammals’ milk except butter), and non-dairy animal protein (mostly meat and poultry, and also fish and eggs). The total amount of protein from several food items which contained more than one source of protein (such as mixed dishes, e.g., pizza, pie) was distributed between the categories, according to the relative contribution of each protein type to the total amount. The total intake of dietary protein and animal protein was also calculated.

### 2.4. Weight Status Definitions

Participating students were measured for height and weight by study personnel, using standardized tools [25]. BMI z-scores (BMIz) were calculated according to the WHO growth standards [32], and values were presented as SD scores. BMIz were divided into four categories: “underweight” less than −2 SD, “normal weight” ≥(−2) to <1 SD, “overweight” ≥1 to <2 SD, and “obese” ≥2 SD [32]. The outcome measure was the prevalence of “overweight/obesity” which is defined as BMIz ≥ 1.0, compared to “normal weight”.

### 2.5. Statistics

SAS statistical software release 9.4 (SAS Institute, Cary, NC, USA) was used to perform the analyses. Normality tests were performed using the Kolmogorov–Smirnov test. Variables that were distributed normally were reported as mean ± SD, and those that were not as median (IQR). Categorical variables were reported as *n* (%). These tests were evaluated with the application of survey weights (conducted according to sex, population group, and school level). Significance of the differences between overweight/obesity and normal weight categories was tested using the Student’s *t*-test or Mann–Whitney U test for continuous variables and the chi-square test for categorical variables. Logistic regression models were used to examine the odds ratios (ORs) and 95% confidence intervals (95% CIs) of overweight/obesity for each of the following independent variables: (1) total protein, (2) total animal protein, (3) non-dairy animal protein, (4) dairy protein, and (5) plant protein. Multivariable models were carried out, including the following potential confounding variables: age, sex, SES, height z-score, physical activity, and total energy intake [11]. Two-tailed tests were run and *p* < 0.05 was considered significant.

## 3. Results

### 3.1. Sample Characteristics

In total, 3443, 11–18-year-old students (78% Jewish, 52% females) were included in the analyses. Participants’ demographic, health, and nutritional characteristics are presented in Table 1. Their macronutrient intakes were within the Acceptable Macronutrient Distribution Ranges [31]. Total protein intake (median (IQR)) was 67 (50, 90) g/d in males, and 58 (41, 80) g/d in females. Overweight/obesity was observed in 31.4% of participants, 33% of males, 29.9% of females. Overweight/obese adolescents were younger, had higher height z-scores, and were more likely to have a lower SES, compared to those with normal weight. Reported median energy intake was slightly lower in overweight/obese youths, however, prevalence of physical activity at recommended levels was similar between groups.

### 3.2. Protein Intake and Weight Status

Daily total protein and protein intake categorized by source (plant or animal) are presented in Table 1. Adolescents with overweight/obesity had higher consumption of energy from all sources of protein (total protein), along with more energy from animal protein and non-dairy animal protein than participants with normal weight (all *p*-values < 0.05). No differences between groups were found in the consumption of energy from plant or dairy proteins.

**Figure 1:**
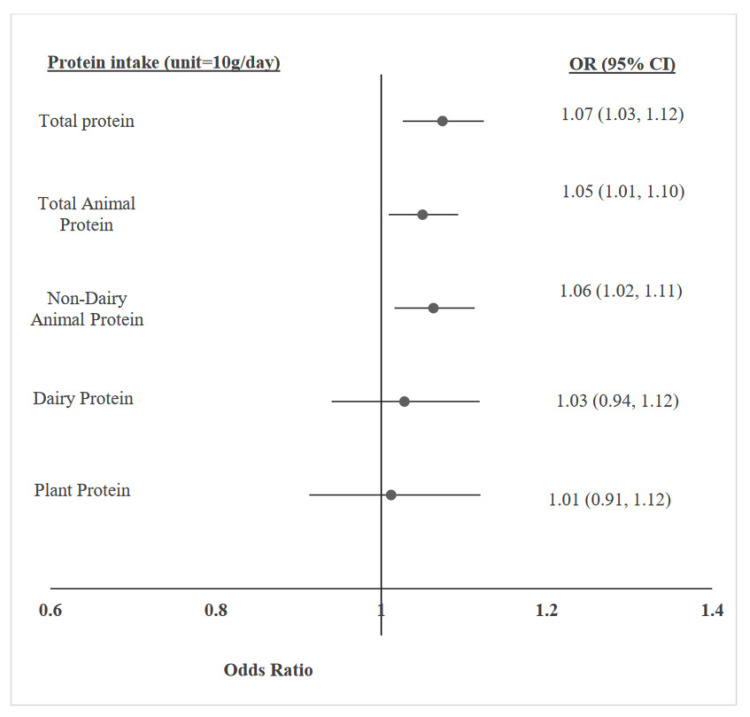
Multivariable logistic regression models, adjusted for age, sex, height z-score, socioeconomic status, physical activity, and total energy intake, for the association between protein intake (g/day)—in total and by source—and adolescent overweight/obesity.

The association between protein consumption (total/by source) and overweight/obesity were evaluated using multivariable logistic regression analyses, controlling for potential covariates, including age, sex, height z-score, SES, physical activity, and total daily energy intake. Incremental increases of 10 g/d in total protein, total animal protein, and non-dairy animal protein were significantly associated with 7%, 5%, and 6% higher prevalence of overweight/obesity, respectively. No associations were found with plant or dairy protein (Figure 1). These positive relationships remained significant when protein intake was reported as percentage of daily energy. Each 2.5% increment in the intake level of total protein, animal protein, and non-dairy animal protein was associated with a higher prevalence of overweight/obesity, with odds ratios (95% CI) of 1.16 (1.08, 1.26), 1. 12 (1.05, 1.19), 1.12 (1.04, 1.21), respectively. Separate tests for overweight and obesity yielded similar findings (Table 2).

## 4. Discussion

In this large national cross-sectional study, protein intake, largely from non-dairy animal sources, was significantly associated with overweight and obesity in adolescents. These results are in line with previous epidemiological studies. The positive association between protein intake and excess weight has been demonstrated predominately in data from infants and children (<12 years of age) [18,33,34]. Several studies investigated these associations in adolescents. An increased intake of energy provided by total and animal proteins was positively associated with overweight/obesity in 12–18-year-old Seventh Day Adventists [24], and with BMIz of adolescents from Germany [35]. In another population-based dietary survey in European adolescents (12.5–17.5 years), animal protein intake was associated with higher BMIz and fat mass [23]. Associations between diet and high fat mass were also investigated in Dutch adolescents and young adults, aged 12–28 years. A 1.5 significantly higher risk of elevated fat mass with higher daily energy intake from protein was reported [36]. In agreement with the present study, there was no positive association between body weight or composition and plant protein consumption in other studies [23,24,35,36], whereas several studies, mainly those conducted in adults, noted an inverse relationship between plant protein intake and risk of obesity [37,38,39].

In the present study, no association was found between protein derived from dairy products and overweight/obesity. Similar findings were observed in prospective investigations in the EPIC study, which included European adults. It was shown that animal protein from red and processed meat, as well as poultry, was associated with increased weight, whereas protein from fish and dairy sources did not correlate with any weight changes [16].

The stimulating effect of total protein and different protein sources on increased body weight is not clear and needs further investigation. It has been suggested that protein intake, especially from animal sources, could play a role in the development of body adiposity through enhancement of stimulation of the bioactive peptide insulin-like growth factor-1 (IGF-1). IGF-1 has a strong anabolic effect on growing adipose tissue [40] in addition to promoting bone tissue development [41] during periods of growth. In contrast, a plant-based diet was associated with lower IGF-1 compared to meat-containing diets [42]. This finding could explain the lack of association between plant protein and overweight/obesity in the current study. In addition, emerging research indicates that higher levels of certain obesity-related gut microbiota are positively related to animal protein consumption [43]. Another possible explanation for the associations between animal protein, especially non-dairy animal protein, and high body weight is the intake of red meat, and processed meat and poultry (main sources of non-dairy animal protein in our study). These animal protein sources contain large quantities of fat and saturated fat. It has been proposed that the combined intake of animal protein and fat may accelerate insulin resistance and has an adverse effect on body adiposity [44]. Moreover, it has also been suggested that a high intake of red and processed meat may reflect consumption of other obesity-related components of the Western dietary pattern such as fast food, sugar-sweetened beverages, butter, and refined grains [45].

It is also possible, as shown in Dutch youth [46], that consumption of protein from dairy sources may be a proxy indicator for healthier eating patterns among adolescents, characterized by a more balanced diet, with higher intake of vegetables, fruits, and whole grains [46]. In addition, dairy sources might prevent weight gain due to the high content of calcium and branched-chain amino acids (BCAAs) which may function synergistically [47] and play a role in the modulation of body fat during developmental ages [48].

The study has some limitations. The cross-sectional design of the survey can only establish associations and could not determine causal relationships. Additionally, despite the widespread use of the FFQ for collecting dietary data, it is subject to social desirability response bias. It is of interest to look at the different subgroups of non-dairy animal protein sources. However, the FFQ data do not allow for separation of meat (beef) and poultry consumption, and very low reported intake of eggs and fish (<0.5% of daily energy intake) did not allow for individual evaluation of these protein sources. Lastly, measurements of body fat mass or lean body mass would have been desirable but were not available for this study. However, BMI, which was used in this study, is a simple anthropometric measure that has been widely and routinely used to identify overweight and estimate body fat [49]. A major strengths of this study was the large representative sample of adolescents. Moreover, anthropometrics (height and weight) were measured by trained personnel, using standardized tools, and SES classifications were used based on the Israeli Ministry of Education data.

## 5. Conclusions

Intake of dietary protein, predominately from non-dairy animal sources (mostly meat and poultry), was positively associated with overweight/obesity among Israeli adolescents, whereas no such associations were observed for plant and dairy protein consumption. The current study adds invaluable information to the limited evidence regarding the associations between consumption of different sources of protein and overweight/obesity among adolescents. It highlights that plant protein and different subtypes of animal protein may play different roles in the development of obesity. Prospective and intervention studies are warranted to further investigate the causal relationship of protein, particularly different sources of protein, with overweight/obesity in adolescents.

## Figures and Tables

**Table 1:** Demographic, health, and nutritional characteristics of study participants according to weight status ^1,2^.

Variable	All (*n* = 3443)	Normal Weight (*n* = 2339)	Overweight/Obese (*n* = 1104)	*p*-Value
***Demographics ***				
Age, *y*	15.2 ± 1.6	15.3 ± 1.6	15.1 ± 1.6	<0.0001
Male sex, %	47.8	46.7	50.3	0.032
Low socioeconomic status, %	32.8	32.1	34.3	0.08
***Measurements***				
Height z-score, *SD*	−0.05 ±1.01	−0.07 ± 0.02	0.02 ± 0.03	<0.0001
BMI z-score, *SD*	0.50 ± 1.06	−0.06 ± 0.01	1.73 ± 0.02	<0.0001
Physical activity as recommended, %	31.6	31.3	32.2	0.11
***Nutritional intake***				
Energy, *Kcal/d*	2105 (1534, 2867)	2144 (1553, 2949)	2040 (1502, 2719)	0.002
Carbohydrates, %Kcal	54.4 (50.3, 58.6)	54.7 (50.8, 58.9)	53.5 (49.1, 57.9)	<0.0001
Fats, %Kcal	31.3 (28.3, 34.0)	31.1 (28.2, 33.9)	31.6 (28.5, 34.4)	0.004
Total protein, *% Kcal*	12.0 (10.5, 13.6)	11.9 (10.4, 13.3)	12.1 (10.8, 14.0)	0.001
Total animal protein, *% Kcal*	6.6 (4.9, 8.4)	6.5 (4.8, 8.3)	6.8 (5.1, 8.9)	0.005
Dairy protein, *% Kcal*	2.5 (1.6, 3.5)	2.5 (1.6, 3.4)	2.5 (1.7, 3.6)	0.18
Non-dairy animal protein, *% Kcal*	3.7 (2.5, 5.3)	3.7 (2.5, 5.2)	3.9 (2.5, 5.5)	0.037
Plant protein, *% Kcal*	5.2 (4.5, 5.9)	5.2 (4.6, 5.9)	5.2 (4.5, 6.0)	0.57

^1^ Percentages calculated with application of weights from the 2nd Israeli Youth Health and Nutrition Survey; ^2^ data are mean ±SD, median (IQR: 25%, 75%) unless otherwise indicated. *p*-values for the differences between overweight/obesity and normal weight.

**Table 2:** Logistic regression analyses of the association between intake of total protein and protein from different sources with overweight and with obesity in study participants *.

	Overweight (1 ≤ BMI z-Score < 2)		Obese (BMI z-Score ≥ 2)	
**Protein Intake, % Daily Energy** *(unit = 2.5%)*	**OR (95% CI)**	***p*** **-Value**	**OR (95% CI)**	***p*** **-Value**
Total protein	1.14 (1.05, 1.25)	0.01	1.22 (1.08, 1.38)	0.01
Total animal protein	1.10 (1.02, 1.18)	0.01	1.17 (1.06, 1.30)	0.01
Dairy protein	1.08 (0.95, 1.24)	0.24	1.15 (0.95, 1.40)	0.15
Non-dairy animal protein	1.10 (1.01, 1.20)	0.02	1.18 (1.04, 1.33)	0.01
Plant protein	1.02 (0.86, 1.22)	0.81	0.91 (0.70, 1.18)	0.47
**Protein intake** **grams/day** *(unit = 10 gr)*				
Total protein	1.06 (1.01, 1.11)	0.02	1.11 (1.03, 1.20)	0.01
Total animal protein	1.04 (0.99, 1.09)	0.07	1.09 (1.02, 1.16)	0.01
Dairy protein	1.02 (0.93, 1.13)	0.63	1.04 (0.91, 1.20)	0.55
Non-dairy animal protein	1.05 (0.99, 1.11)	0.07	1.10 (1.02, 1.18)	0.01
Plant protein	1.03 (0.92, 1.16)	0.6	0.96 (0.81, 1.14)	0.65

* Adjusted for age, sex, height-z-score, socioeconomic status, daily energy intake, and physical activity.

## Data Availability

Israel Center for Disease Control provides data upon request and according to its procedures.
